# Challenges in current inhalable tobacco toxicity assessment models: A narrative review

**DOI:** 10.18332/tid/188197

**Published:** 2024-06-10

**Authors:** Thivanka Muthumalage, Alexandra Noel, Yasmin Thanavala, Aleksandra Alcheva, Irfan Rahman

**Affiliations:** 1 School of Health Sciences, Purdue University, West Lafayette, United States; 2 School of Veterinary Medicine Louisiana State University, Baton Rouge, United States; 3 Department of Immunology, Roswell Park Comprehensive Cancer Center, Buffalo, United States; 4 Division of Environmental Health Sciences, School of Public Health, University of Minnesota, Minneapolis, United States; 5 Masonic Cancer Center, University of Minnesota, Minneapolis, United States; 6 Department of Environmental Medicine, University of Rochester Medical Center, Rochester, United States

**Keywords:** electronic cigarettes, vaping, heated tobacco products, inhalation toxicology, *in vitro* and *in vivo*

## Abstract

Emerging tobacco products such as electronic nicotine delivery systems (ENDS) and heated tobacco products (HTPs) have a dynamic landscape and are becoming widely popular as they claim to offer a low-risk alternative to conventional smoking. Most pre-clinical laboratories currently exploit *in vitro*, *ex vivo*, and *in vivo* experimental models to assess toxicological outcomes as well as to develop risk-estimation models. While most laboratories have produced a wide range of cell culture and mouse model data utilizing current smoke/aerosol generators and standardized puffing profiles, much variation still exists between research studies, hindering the generation of usable data appropriate for the standardization of these tobacco products. In this review, we discuss current state-of-the-art *in vitro* and *in vivo* models and their challenges, as well as insights into risk estimation of novel products and recommendations for toxicological parameters for reporting, allowing comparability of the research studies between laboratories, resulting in usable data for regulation of these products before approval by regulatory authorities.

## INTRODUCTION

In a continuously evolving tobacco product landscape, the availability of combustible tobacco products, such as cigarettes, cigars, and waterpipe or hookah, and non-combustible tobacco products such as electronic nicotine delivery systems (ENDS) or electronic cigarettes and heated tobacco products (HTPs), is rapidly increasing. These novel non-combustible products are available in tobacco-derived (TDN) and tobacco-free nicotine (TFN) forms, but due to the sparsity of data on toxicity, health effects and increased consumption, these products have become a significant public health concern. Various TFN e-juices for vape pens and heat sticks for IQOS (e.g. LEVIA) are emerging synthetic products evading tobacco regulations. These products and devices have been evolving and diversifying in their features^1-3^. Some ENDS resemble traditional cigarettes, pens, or flash drives, while others are complex systems with large tanks, heating elements, batteries, and customizable parts or power settings. Versatility in the product design features and variations in use parameters, including power (wattage) and heat customizability influencing inhalation topography, result in the exposure of the users to different aerosol chemical profiles^4,5^. Constituent analyses of smoke or aerosols from these products integrated with cell culture and animal studies to identify biomarkers of human exposures and disease, have provided initial insight into their potential toxicity^6-8^.

Studies have shown that the aerosol from non-combustible products are associated with altered immune responses, inflammation, and oxidative stress responses similar to combustible products^9-22^. Given the risk of health effects on the respiratory, cardiovascular, and systemic effects, many studies have used cell lines, primary cell cultures, and animal models representing target organ toxicity.

The current knowledge base indicates the considerable toxicity potential of inhalable nicotine and tobacco products. Limitations in research methodology and the wide range of variables involved in studying the effects of these products *in vitro* and *in vivo,* pose challenges to the scientific and regulatory communities. Current risk estimation approaches rely on comparative assessments of combustible smoke, marginalizing the modified risk posed by the emerging products^23,24^. Standardization of exposure paradigm across laboratories is needed for consistent and comparable toxicity and risk estimation of tobacco products. Standardizing exposure and toxicity parameters *in vitro* and *in vivo* studies will improve their translational relevance to human exposure and toxicity. Our goal in this review is to summarize the approaches and methodologies used in *in vitro* and *in vivo* studies to date, and to identify factors that could be improved in designing and reporting the relevant outcomes of these studies when comparing toxicity across emerging tobacco products. Recommendations here could further support and enhance the tobacco regulatory science framework and strengthen current tobacco control efforts.

## CURRENT MODELS FOR INHALATION TOXICITY ASSESSMENT OF TOBACCO PRODUCTS

### Exposure regimens

Research in tobacco regulatory science focusing on investigating the health effects and toxicity of inhalable tobacco products, faces many challenges. Two of those challenges are: 1) reproducibility of the data, and 2) comparison of the data between exposure systems and laboratories. When conducting experimental studies aimed at simulating real-life exposures to inhalable tobacco products, including combustible cigarettes, ENDS, waterpipe smoke, and HTP, it is critical to use exposure conditions that are representative of human users’ behavior. This is a first step in improving the translational impact of the research. Indeed, the smoking or vaping topography, including puff regimes and profiles, are key factors to consider when designing and conducting both *in vitro* and *in vivo* experiments.

**Figure 1:**
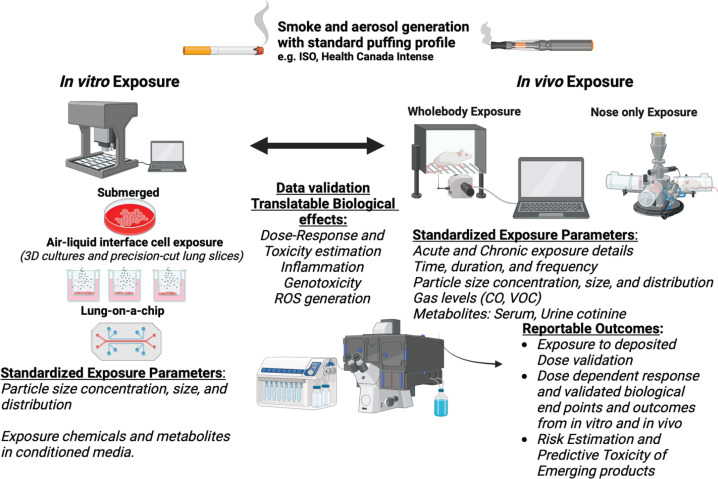
Summarized *in vitro* and *in vivo* state-of-the-art methods

In general, smoke and aerosols are generated according to approved puffing regimens, such as those from the Federal Trade Commission/International Standard Organization (FTC: ISO), Health Canada (HC), and the Massachusetts Department of Health (MDO) (Figure 1). These regimens depend on pressure drop, and puff duration, puff volume, puff number, and puff frequency. Most commercial cigarette smoke and aerosol generators are pre-programmed with these acceptable puffing profiles. Both International Organization for Standardization (ISO) and Cooperation Centre for Scientific Research Relative to Tobacco (CORESTA) are available resources for tobacco and related tobacco products when developing exposure studies. For example, ISO compliance includes, 35 mL puff volume, 2 s puff duration, and 60 s inter puff interval^25^. Whereas in the HC intense (also now ISO intense profile, compliance includes, 55 mL puff volume, 2 s puff duration, 30 s inter puff interval)^26^. Further, adaptations of these profiles are sometimes seen in ENDS aerosol generation regimens with puff volumes ranging from 45–70 mL and puff duration of 1.8–4.0 s. The CORESTA Recommended Method No 81 (CRM 81) or equivalent ISO 20768 (vaping regime: puff volume, 55 mL; puff frequency, per 30 s; puff duration), is a vaping profile commonly used in ENDS toxicity studies^27^. The greatest hurdle of smoke/aerosol topography profiles is that no protocol can represent all human smoking or vaping behavior^28^. Therefore, for emission characterization and toxicity studies, it is imperative to maintain consistent puffing profile throughout the experiment. In addition, for secondhand smoke generation, usually mainstream (15%) and sidestream (85%) smoke are mixed with HEPA-filtered air in a dilution chamber. Atmosphere for conditioning of tobacco products, for example, 48 h using a forced air flow for loose cigarettes, is recommended (ISO 3402) as combustion of tobacco varies based on the conditioning of the cigarettes. Further, there is a Beirut-based waterpipe (hookah) smoking regime: puff volume, 530 mL; puff frequency, per 15.48 s; puff duration: 2.6 s) ^29^, which depends on the type of tobacco product studied. Overall, using these standardized topography parameters for a specific inhalable tobacco product is key for reproducibility and comparison between exposures and studies.

A number of factors, including topography, affect the ENDS aerosol physicochemical characteristics affecting reproducibility and comparability between laboratories^5,30^. These other factors include the e-liquid composition (humectant ratios, propylene glycol [PG], glycerin [G] or vegetable glycerin [VG], flavoring chemicals, nicotine concentration, and other additives), device type (open airflow and closed systems), type of coil used (resistance, type of metal, wick composition), power (wattage, voltage), and heating conditions (temperature). Of important consideration is the heating temperature of the e-liquid, which will influence both the chemical (e.g. presence and concentration of carbonyls) and physical (e.g. droplet number and size) profiles of the ENDS aerosols. All the factors mentioned above, which are innate to the device used, alter the primary emission constituents, pyrolytic, and secondary emission in both aerosol and gas phases. In addition, the presence of nicotine and flavoring chemicals, along with their concentration in the e-liquid, will further affect the resulting aerosols. Hence, a significant challenge related to the toxicity assessment of ENDS is due to the rapidly evolving ENDS market, which creates a broad spectrum of possible combinations of these factors and ENDS-related parameters, each potentially producing a unique toxicity. Thus, although difficult and complex, keeping these factors consistent from one experiment to the next will help maintain consistency and allow comparison of studies from different laboratories. Similar to the combustible reference cigarettes by the University of Kentucky Center for Tobacco Reference Products, the National Institute of Drug Abuse (NIDA) has developed a standard electronic cigarette to be used in research settings. However, this NIDA standardized device (SREC) could become rapidly obsolete as new generations of ENDS devices continually appear on the market. This may be why only a few studies have reported its use thus far^31-38^.

Heated tobacco/heat-not-burn cigarettes are an alternative to traditional combustible cigarettes that involve heating tobacco sheets instead of burning ground tobacco leaves^39^. While conventional cigarettes produce toxicants through the process of combustion, heated tobacco products primarily follow the thermal degradation of substrate constituents. These products emit less particulate matter, but these devices still produce concerning levels of lung irritants and toxic chemicals that could impact respiratory health. Composition analyses have shown that heat-not-burn cigarettes generate compounds like acrolein, formaldehyde, and benzene, which are hazardous to the lungs^40^. Chronic and acute *in vivo* studies have shown similar inflammatory responses to combustible cigarette smoke and emphysema phenotypes in mice^14,41^. Both studies stated serum cotinine levels, as an exposure biomarker to tobacco, of 29.5 ng/mL and 300 ng/mL, which are greater than a non-smoker level of <1 ng/mL. Further, Bhat et al.^29^ stated the PM2.5 concentration (about 197 µg/m^3^) and atmospheric conditions, including nicotine levels, which are critical parameters of an animal exposure study for translational relevance and reproducibility^29^. More studies are needed to estimate the risk of heated tobacco products to understand the chronic effects of heated tobacco.

As illustrated here, experimental conditions for inhalable tobacco products can differ widely based on the selection of the smoking/vaping topography as well as the choice of tobacco or vaping products. In order to have a more complete understanding of the toxicity and health effects of inhalable tobacco products, there is a clear need for the scientific community to standardize experimental protocols, particularly for users’ topography. All parameters selected for a specific study should be justified, relating to real-life exposure levels, recorded, and reported in the resulting publication. This will be beneficial for the comparison of studies evaluating similar tobacco products being conducted by different research groups.

### *In vitro* toxicity testing models

Although various inhalable tobacco products, including cigarettes, ENDS, HTPs, and hookah, have established or recommended puffing topography profiles to be used in research (cf. section above), there are currently no standardized protocols for the use of those inhalable tobacco products when assessing *in vitro* toxicity. This is even though regulatory and research agencies have never been more encouraging toward *in vitro* testing to reduce animal studies in biological experiments. Studies evaluating the *in vitro* effects of aerosols or smoke are often performed in submerged or under air-liquid interface (ALI) conditions. As is expected for any model, cell culture experiments inherently have factors that need to be considered for reproducibility, such as choosing the most appropriate cell type/line, passage of the cells, confluency of cells during treatment, serum deprivation, treatment dose, duration, and handling techniques. Meticulous and consistent adherence to these parameters are quintessential for achieving a successful and robust cell culture experiment.

Inhalable tobacco products enter the airways via the respiratory epithelium, which is the first physical barrier at the junction of host–external environment interactions^42^. The selection of the cell type will mainly rely on the scientific question being addressed by the study; however, both primary cells and cell lines can be used, with primary cells more closely mimicking *in vivo* organs and representing greater translational impact value. Primary cells can be affected by variation between donors as well as by cell passage used^43,44^. Transformed, cell lines have a longer life span, with much less variability between passages, and therefore can be used for long-term exposure study to inhalable tobacco products ^43-46^. Cells can come from the various regions of the respiratory tract, including tracheal, bronchial and alveolar epithelial cells^47^. Co-cultures can more accurately represent the lung environment compared to the use of monocultures^48^. While conducting *in vitro* experiments, the use of cancer cell lines should be avoided since they may not exhibit normal physiological behaviors, including increased sensitivity or tolerance to a particular compound^49^. In addition, in exposure models, the selection of the exposure dose (e.g. total particulate matter/particle size distribution) is important and must reflect the expected internal dose at the target organ as there is particulate loss after combustion/aerosolization to the deposited dose. Selecting exposure doses that are much higher or lower than the average exposure to a consumer by a product, can impact the cellular responses and induce an *in vitro* response that greatly differs from what would be seen *in vivo* models or in humans^49,50^.

Some studies use cigarette smoke or ENDS aerosol extracts in submerged cell culture treatments to assess biological responses and cytotoxicity. When preparing cigarette smoke extract, standardization of the extract is important. The same exact brand, type of cigarette, and lot number need to be used throughout the experiment since the composition of the cigarette varies. Smoke extract preparation includes bubbling smoke through media using an impinger. The flow rate of bubbling, the number of cigarettes used, mixing and aging of the solution, and the pH of the solution are key factors determining the biological effects induced at the selected treatment concentration. Standardization of these aqueous extracts for batch-to-batch can be achieved by measuring the nicotine concentration of the solution by gas chromatography and constant optical density for nicotine/tar (260–320 nm), and determining the appropriate treatment concentration depending on the cell type by performing appropriate dose-response relationships^51,52^.

It is essential to bear in mind that cigarette smoke and e-cigarette aerosols are composed of both particulate and gas phases^51,53-56^. Due to the complexity of these smoke and aerosol mixtures, it is difficult to expose cells to both phases simultaneously when using traditional submerged cell culture conditions. The use of cigarette smoke or e-cigarette aerosol extracts (representing the aqueous components) or condensates (representing the lipid soluble components) only captures a finite fraction (aqueous or lipid soluble) of these complex mixtures^56,57^. Although extracts and condensates of inhalable tobacco products represent tools that can be used efficiently and have very high reproducibility in toxicological testing, they should not be the standard for *in vitro* experiments, as they may not accurately reflect (e.g. underestimate or overestimate) the cellular responses to the complex mixture of the smoke or the aerosol as a whole.

On the other hand, ALI cell culture models provide many advantageous characteristics over traditional submerged cell culture conditions regarding reproduction of *in vivo* pulmonary host-defence interactions, lung physiology and accurate measurement of deposited dose^10,12,58-63^. For instance, bronchial epithelium grown and differentiated into stratified cells (basal cells, goblet cells, ciliated cells) at the ALI develop cilia, can form tight junctions, and can release mucin as well as pro- and anti-inflammatory mediators^64,65^. This allows for recreating human lung physiological interactions more closely. In ALI conditions, however, the puffing profile, number of puffs, dimensions of the culture wells, length of tubing carrying the smoke/vapor, temperature, and humidity are some of the factors that impact the physicochemical properties of the aerosol and ultimately the biological responses induced. The state-of-the-art instruments available currently provide ALI conditions representing physiological gas exchange and maintaining 37°C at the exposure site. However, the tubing lengths, surface area, and exposure chamber dimensions are not identical from one system to another. In addition, most currently available *in vitro* exposure systems do not precisely simulate the changes during inhalation of smoke aerosols. As smoke/vapor enters nasal passages, it is humidified and warmed to 37°C, then travels through the trachea, lower respiratory tract, and alveolar regions. The hygroscopic properties of the droplets from the aerosols and the humidity encountered in the respiratory tract will influence the physical characteristics of the aerosols once they reach the lower airways. For *in vitro* experiments, cell or tissue type and de-identified donor information, including donor’s age, sex, race, prior environmental exposures, and medical history must be reported.

Moreover, ALI exposures are time-consuming, and replicates can be limited due to the cost and the design of the exposure module^44,66^. Thus, conducting high throughput exposures can be a challenge. Therefore, some researchers use homemade ALI instruments to obtain *in vitro* data, making comparison of results even more challenging, as the puffing profiles and exposure conditions may not be identical, leading to changes in aerosol composition, characteristics, and biological responses. Protocols for ALI cell differentiation, maintenance, and exposure to inhalable tobacco products are not standardized and thus vary greatly between research groups and represent a current limitation in tobacco regulatory science research.

In addition, when cells are grown and differentiated at the ALI, aerosolized tobacco products in the form of smoke or aerosol can be directly deposited at the surface of the cells. The deposited mass at the ALI can be measured (estimated) using a crystal quartz microbalance, while the amount of smoke or aerosol deposited when using submerged conditions is challenging to estimate. Moreover, the chemical species present in both the particulate and gas phases of the smoke or aerosol will interact with the cells grown at the ALI. This realistic component cannot be recreated in submerged conditions. Therefore, substantial differences between estimated exposure versus deposited doses for submerge versus ALI conditions, may exist. All these variables lead to challenges when comparing the *in vitro* toxicity of inhalable tobacco products. It was previously demonstrated that the exposure of lung cells at the ALI to whole cigarette smoke was a better model to recapitulate levels of nicotine/cotinine measured in the sputum of smokers than submerged *in vitro* models using cigarette smoke condensates^56^.

It is currently unclear whether *in vitro* experiments on inhalable tobacco products are more reliable when conducted at the ALI or under submerged conditions using extracts or condensates^67^. For the effects of cigarette smoke, the majority of *in vitro* experiments have been conducted on extracts and condensates^67-70^, while few studies have been conducted at the ALI^71-75^. When it comes to ENDS aerosols, an increasing number of recent studies on ENDS aerosols use ALI exposure conditions^10,16,51,76^. When comparing the toxicity of ENDS products using ALI versus submerged exposure conditions, overall, biological endpoints seem to follow similar tendencies; for instance, IL-8 is increased following ENDS exposures, whether in the form of aerosol or extracts ^9-12,16,61^. Whether one experimental condition is more sensitive than the other is currently unknown. ALI conditions may yield more accurate biological data than submerged conditions and, therefore, provide more reliable data when doing extrapolations for human risk assessment^77,78^.

Recently, organ-on-a-chip models such as lung-on-a-chip and multi-organ-chip models have gained popularity as alternative methods to animal testing for the characterization of smoke and vapor-exposed lung injury. These models emulate structural, functional, and mechanical properties of the alveolar-capillary interface, and epithelium-endothelium interface, and range from lung, heart, liver, and more^79-81^. The lung-on-a-chip and multi-organ chips are valuable in toxicity testing, drug screening, metabolism profiling, pharmacokinetics analysis, and human disease modeling. Organ-on-a-chip models have advantages over animal models, such as predicting human-specific disease modeling of a particular organ but marginalize systemic effects ^81-86^. Microfluidic organ chips can recapitulate multi-tissue interactions and responses such as tissue expansion stress. Despite the advantages, there are still challenges with these models related to the design^87,88^. The current tobacco and ENDS generators targeted for ALI models are not entirely compatible with the chip models conforming to normal lung physiology. Other *in vitro*/*ex vivo* models include precision-cut lung slices (PCLS), often used with intact alveoli. Crucially, human and mouse *ex vivo* PCLS are increasingly used in toxicity testing studies and lung biology. Similarly to ALI, in PCLS, donor age-sex matching is crucial for reproducibility. Very few studies have used PCLS methodologies to assess the pulmonary health effects of inhalable tobacco products, hence it is too early to determine the appropriateness of these methods to assess the health risk associated with the use of emerging tobacco products.

Overall, *in vitro* experimentations are advantageous as they allow for mechanistic studies due to controlled uniformity of the treatments and less confounding factors compared to animal models. They allow for early detection of modes of toxic action and adverse effects of inhalable tobacco products^89,90^. One drawback of *in vitro* studies is that establishing the long-term toxicity of tobacco products is not feasible when using these models^49,91^. Biological outcomes for *in vitro* samples may include those obtained by ‘omics’ technologies, such as genomics, transcriptomics, proteomics, and metabolomics responses. Further integrated system biology approaches offer wide-ranging overviews of changes occurring at the cellular and molecular levels following exposure to an inhalable tobacco product. Moreover, system biology data are an asset that can be used to correlate effects observed in both *in vitro* and *in vivo* models^92^. In summary, for *in vitro* studies, performing appropriate toxicological characterization of the smoke/aerosol, including mass deposited, dose and particle size distribution (Total particulate matter (TPM)/particulate matter (PM) concentration and particle size), should be recorded and reported. Other parameters to record and report include dilution and flow rates, in-line quality control methodologies used to determine dose delivery and consistency of dose, as well as key constituents, such as nicotine and total volatile organic compounds concentration.

### *In vivo* toxicity testing models

A number of parameters, will affect the overall results in experiments designed to elucidate the effects of tobacco smoke and ENDS aerosols using preclinical animal models. Thus, puffing profile, exposure dose, duration of exposure, frequency of exposure, temperature, humidity, exposure systems, battery power, flavoring, product brands, animal species, animal age, animal sex, and animal numbers are all important when assessing the potential effects of smoking and vaping on human health using *in vivo* animal models. It is also important to know if the experimental conditions are designed to mimic light, medium, or heavy smokers or an experienced or novice vaper. The use of standardized reference tobacco cigarettes versus commercially available cigarettes may also introduce a factor of variability in experimental outcome, as will the flavoring component of ENDS. An overall variable to note is that the plasma half-life of nicotine differs in rodents versus humans but has been generally estimated following i.v. or i.p. nicotine delivery, not following inhalation exposure. These variables, therefore, underscore an urgency to provide transparent descriptions of parameters utilized and how they may affect risk evaluation.

The two most commonly used models, for *in vivo* exposure studies in rodents, to examine the effects of smoking and vaping, rely on systems designed to allow nose-only (NO) or whole-body (WB) exposures to smoke or ENDS aerosols. It is important to compare the advantages and potential limitations inherent to both systems. A merit of the NO systems is, because of the small chamber volume, there is less material waste and there is avoidance of exposure via routes other than inhalation. On the flip side, there is the serious concern that animals need to be kept immobilized/restrained for the duration of exposure, resulting in the generation of stress to the animal^93,94^. The stress will potentially affect the study readouts. Mice of different strains, ages and sex may also differ in their level of sensitivity to restraint. This may be reflected in measures of body weight changes, observations of tremors coupled with higher nicotine concentrations in plasma. Due to the considerably smaller size of the chamber in the NO exposure system, the saturation and equilibrium may likely be reached more rapidly. There is also the issue that it is generally difficult to control humidity in this system.

In contrast, in the WB exposure system, the animals are not restrained and move freely within the exposure cages, and thus, there is the avoidance of imposed stress. The WB systems are, therefore, considered more suitable for long and repeated exposure experiments. However, there needs to be consideration that exposure from routes besides inhalation of the test material could impact the results. Thus, the deposition of particulate material on the fur of the animals or the chamber walls must be considered and experimentally accounted for when determining total exposures. The use of bedding, nesting paper and plastic material in cages may also influence the deposition of material inside the WB chamber. Generally, because of their volume differences, NO and WB exposure chambers require significantly different aerosol flow rates, making it challenging to perform strictly comparative studies using the same experimental design. The suitability of the choice of exposure system will also be dictated to some extent by the tissue/organ site being studied. Thus, differences have been noted in the severity of effects in the nose when using WB versus NO exposures, depending on the disease phenotype and organ system studied^95-98^. Despite these differences, there are reports where comparison studies have been performed with informative data generated^10,13,14,17,19,99-108^. One way around the conundrum may be to aim to achieve comparable aerosol characteristics for exposures, by matching TPM concentration with particle size distribution. Another approach is to equalize the blood cotinine concentration achieved by the different exposure protocols.

Gases and vapors are distributed throughout the airways upon inhalation. In contrast, particle concentration will vary, and their pulmonary deposition will depend on particle size, particle density, airflow and respiratory tract anatomy of the animal species being utilized. Particles may be retained at site of deposition or there may be cleared by mucociliary activity. It is known and must be recognized, that the morphology of the respiratory tract is different between humans and rodents. In the case of inhalation studies, humans inhale via the mouth or the nose, and rodents via the nose only. A large variable in the published literature arises from the duration of the exposures, irrespective of the type of chambers used. Thus, exposures range from 30 minutes to weeks or months, with a single exposure session or multiple exposures per day, with intermittent exposure versus continuous exposures. These differences can pose a challenge to data interpretation as well as the induction of different biological outcomes.

Overall, whether exposures are conducted via NO or WB exposure systems, the TPM concentration resulting from the smoke or aerosol generation must be measured in the breathing zone of the test animals or inside the exposure chamber and recorded, along with the temperature and humidity. Levels of nicotine inside the exposure chambers are very informative; however, if nicotine concentrations cannot be measured in the smoke or aerosols, levels of nicotine should be measured in the test subjects; for example, nicotine/cotinine levels could be measured in serum or plasma of rodents for *in vivo* experiments.

The current state-of-the-art *in vitro* and *in vivo* models and their relationship in delivering translatable data for regulatory purposes are summarized in Figure 1.

## RELATIVE RISK CHARACTERIZATION OF INHALABLE TOBACCO PRODUCTS

Investigating the toxicological effects of tobacco products also includes the concept of tobacco harm reduction, which signifies ‘minimizing harms and decreasing total mortality and morbidity, without completely eliminating tobacco and nicotine use’^109^. The premise of tobacco harm reduction is to offer cigarette smokers who are unable to quit smoking, nicotine delivery systems that produce less harmful chemicals, enabling the user to continue their nicotine exposure when switching entirely to the alternative product, and ultimately, reduce the occurrence of tobacco-related diseases^110^. Even though harm reduction, based on scientific evidence, should be clear and significantly distinct, this area is still heavily debated in the scientific community^111^. Nonetheless, developing a unified discourse on tobacco harm reduction and relative risk is of importance for healthcare providers, enabling them to make sciencebased recommendations, and for cigarette smokers to make informed decisions^111,112^. Public health authorities in the United Kingdom state that using ENDS is approximately 95% less damaging than using combustion cigarettes^112^. The scientific evidence supporting this statement is unclear; however, some studies conducted by tobacco companies showed reduced toxicity in terms of aerosol chemistry, *in vitro* and *in vivo* data, when comparing e-cigarette aerosols to cigarette smoke^113-117^. Questions to consider are: ‘How do these alternative inhalable nicotine delivery systems prevent dual or poly use of tobacco products, as seen in the real world?’ and ‘Is there a relative risk modification after completely switching from cigarettes to an alternative inhalable nicotine delivery system?’. Currently, the weight of evidence supporting the claims from cigarette and ENDS companies that these alternative inhalable nicotine delivery systems are effective tools to quit smoking are scarce, inconsistent, and limited^118-121^.

One of the crucial questions associated with tobacco harm reduction is ‘how to define a “safer” or “less harmful” alternative product to cigarettes that continues to deliver a satisfactory amount of nicotine to the user and provides an overall reduction in health risk?’. E-cigarettes and heat-not-burn tobacco products are popular examples of such ‘alternative tobacco products’, claimed as ‘safe’ (or less harmful) alternatives to combustion cigarettes by cigarette and ENDS companies. These alternative inhalable nicotine delivery systems are positioned at a lower rank on the risk related to the tobacco-products continuum, as the e-cigarette aerosols and heat-not-burn smoke contain lower levels of toxicants than cigarette smoke^111,112^. This may be true in the context of short-term exposures; however, this beneficial effect of reduced toxicant exposure may not hold true following chronic or prolonged exposures to low levels of those harmful toxicants. Indeed, the range and intensity of effects related to long-term exposures to novel nicotine delivery systems are still unknown. Establishing whether a tobacco product induces reduced harm requires a thorough risk assessment compared to combustible cigarettes. When contemplating tobacco harm reduction assessment, it is essential to bear in mind that: 1) the aerosol chemistry will vary based on the user vaping preferences; 2) *in vitro* data may not reflect the complexity of organ and system biology; 3) *in vivo* models may not recapitulate the human condition in its totality; and 4) human clinical or epidemiological data may not analyze a key tissue of interest due to ethical and invasiveness limitations^122^. Indeed, e-cigarette aerosols and heated tobacco smoke are complex mixtures that contain multiple known and unknown constituents, for which the toxicity following inhalation is not always established^122^. Research in tobacco regulatory science focuses not only on the toxicity of individual components of the aerosols or smoke, but also on the whole aerosols and smoke, for which the effects of the individual components may not be simply additive^122^. The chemical constituents of the ENDS e-liquid, by additive or synergistic interactions, induce adverse responses and toxicity upon heating. Characterizing aerosol/particle and gas-phase constituents of these products is important to developing acute toxicity estimates of the aerosol mixtures. Hence, identifying the key toxicants is essential in comparative toxicity assessments.

Therefore, a tobacco harm reduction risk assessment that would evaluate the toxicity of individual toxicants from e-cigarette aerosols or heat-not-burn smoke, and not consider the interactions or synergy between these chemicals, may overestimate the reduced harm of a tobacco product. While *in vitro* and *in vivo* experimental data can inform on the toxicity of a specific product, the exposure dose and the biologically internal dose differ, making the toxicity estimate challenging. Cell culture models allow the study of organ/tissue-specific biomarkers of toxicity, inflammation, genotoxicity, dysregulated mitochondrial function, and allow for unraveling novel pathways identified in mouse models. In addition, experimental study designs need to include switching behaviors in animal models to better understand the relative risk modification after switching. Confirming and validating exposure-induced responses *in vitro* and *in vivo* help extrapolate the findings with great translational relevance. However, correlating *in vitro* data to *in vivo* data has been difficult due to the lack of standardization in toxicity testing. On the other hand, human data are highly impactful in this context, as reduced levels of clinical risk markers predictive of tobacco-related morbidity and mortality due to the complete switch to alternative inhalable nicotine delivery systems, are the ultimate evidence of harm reduction. Therefore, although very limited, human data carry a greater weight in risk assessment. As expected, each research field has its advantages and disadvantages. Thus, it is essential for the approaches used in tobacco harm reduction assessment to encompass multiple spheres, including chemical profiles of the aerosols or smoke, *in vitro* and *in viv*o toxicological evaluation, clinical trials, and epidemiological studies^111,122^. Further, artificial intelligence (AI) models are emerging in toxicity prediction models. Based on the current databases of composition analyses, biomarkers of exposure and disease, survey data, *in vitro* and *in vivo* data, and existing human data, machine-learning toxicity prediction models can be created for tobacco toxicity outcome models. Taken together, this will lead to a comprehensive scientific assessment of relative harm reduction and potential increased health benefits.

Implementing a regulatory framework in tobacco harm reduction is problematic based on the dynamic tobacco landscape and because several factors can affect the toxicity of the e-cigarette aerosols or heat-not-burn smoke. The presence of four generations of ENDS devices on the market led to the creation of a multitude of ENDS-related variables and combinations of those variables. Indeed, numerous factors can affect the ENDS aerosol toxicity, including but not limited to the type of ENDS device used and the operational ENDS device settings (power) applied, which will influence the temperature at which the e-liquid is heated. These factors including, the puffing topography, and the composition of the e-liquid added to the proportion of the ingredients, as well as the chemical forms of nicotine (free-base or nicotine salt) lead to the thermal degradation of humectants and flavors^4,123-125^. For instance, flavors in e-liquid are mixtures of chemicals that impart a unique aroma and flavor sensation. However, the same flavor sold by different vendors comprises various chemical constituents. Therefore, testing more than one brand of a specific flavor is important for comparative toxicity analysis. When testing a particular product, multiple e-liquids of the same brand and flavor from different batches should be tested to identify batch-to-batch differences in chemical composition of the liquid and aerosol, as well as in toxicity and biological response. This will also allow for the identification of the general toxicological profile of a particular product. *In vitro* and *ex vivo* cultures can be used for high throughput toxicity screening of these chemicals in submerged cultures or by nebulization in *in vitro* air-liquid interface models. Identifying these chemical constituents and the concentrations present in the same flavor sold by various vendors, would create a database for estimated risk assessment between flavors and for predictive toxicity of emerging products. Overall, determining a level of harm reduction for all ENDS devices is extremely difficult, as some open-system third-generation ENDS devices emit carbonyls at levels orders of magnitude higher than those emitted from first, second or fourth-generation ENDS devices^126-130^. At first glance, these data may suggest that some generations of ENDS devices provide a direction of change towards harm reduction; however, even aerosols produced by fourth-generation closed system ENDS devices, which contain less and lower levels of harmful chemicals than cigarette smoke, can induce *in vitro* and *in vivo* toxicity ^10,16,103,131-133^; albeit at a lower degree than cigarette smoke. This clearly demonstrates the important concept that emission reduction, even if it reflects an 80% reduction in the exposure level, does not necessarily translate into 80% safer (less harmful) health outcomes. Together, the complexity of ENDS devices and liquids, suggest that the toxic effects of ENDS aerosols may not be solely due to the toxicants (or carbonyls) emitted from the device but may also be due to the chemical forms of nicotine (free-base nicotine vs nicotine salt) interacting with the other components of the e-liquid. Further, as nicotine binding affinity to receptors can be affected by these forms (e.g. tobacco derived vs tobacco free) this implies that the form/state of nicotine can affect the internal dose and its toxicity. Therefore, these data clearly show that toxicant emissions from ENDS should not be the only criteria to establish harm reduction.

As described above, a growing body of evidence demonstrates that the regulatory framework for tobacco harm reduction should not be based on assumptions and basic extrapolations solely from aerosol chemistry, *in vitro* and *in vivo* data^9,16,29,102,103,126-129,132-135^. Analyzing the toxicity of individual components of the aerosols can be misleading, as it needs to consider interactions. Further, a tobacco product should be tested for its acute, sub-chronic, and chronic toxicity to estimate the relative risk of exposure-related injury. Long-term effects associated with chronic exposures to low doses of a toxicant may be strikingly different from short-term exposures to higher doses of this same compound. Lower exposure levels do not necessarily equate to safe (or less harmful) exposure levels. This is best exemplified by genotoxic carcinogens with no threshold dose for stochastic effects, where there is a risk of adverse effects even following low-dose exposures^136^. Human clinical and epidemiology data are the gold standard despite the long data collection period involved. Overall, when weighing the benefits versus the risk of alternative inhalable nicotine delivery systems in tobacco harm reduction, tobacco regulatory science researchers should not only compare the results to cigarette smoke but most importantly, in a public health context, also include a group that represents abstinence, i.e. a control group that is exposed to clean air, as well as a group representing counterparts switching from combustible products to ENDS^111^.

In summary, when estimating the comparative toxicity and the relative risk of exposure to tobacco products, aerosol chemistry, *in vitro*, *in vivo*, and clinical plus epidemiology data must be used in conjunction. As emphasized throughout this review, harmonization and standardization of methods used across tobacco regulatory science research are crucial not only to compare studies and to draw firm conclusions on the safety and the relative risk of these new alternative inhalable nicotine delivery systems, but also to establish a regulatory framework for harm reduction assessment.

## RECOMMENDATIONS FOR DATA REPORTING

It is important to remember that critical factors related to generating the inhalable tobacco product of interest will impact the delivery of the smoke/aerosol and its toxicity (Figure 1).

For both *in vitro* and *in vivo* studies, it is important:

- To use a standardized topography profile representative of human behavior of different tobacco products throughout an experiment.
- To characterize the aerosol or the smoke and report the key constituents (e.g. nicotine, carbonyls, volatile organic compounds).
- To record and report dilution and flow rates, as well as methodologies used to determine dose delivery and consistency of dose.
- To perform in-line sampling at the site of exposure (breathing zone of the animals) and report nicotine, particle size distribution, and TPM levels.
- To measure and report evidence of nicotine exposure in cells (nicotine cotinine levels in media) or biomarkers (urine serum nicotine/cotinine levels) in animals.

This should improve inter-laboratory comparisons of data. Further, in addition to difficulties when comparing results obtained from different laboratories, with recent increased attention on scientific rigor, reproducibility, even within the same laboratory, can be difficult, thus, intra-laboratory variability also exists. This puts the emphasis on the importance of reporting *in vitro* and *in vivo* exposure conditions as well as a biomarker of exposure for each independent study conducted within a laboratory.

Future needs for standardized research of inhalable tobacco products:

- A universal ENDS device for each generation (reusable and disposable) and heated tobacco products.
- Standardized e-liquids specifically define humectant, flavoring, and nicotine ratios, the form and isomer of nicotine, with stable quality control values across batches and samples.

## CONCLUSION

This non-systematic review has analyzed the current state-of-the-art platforms to assess the *in vitro* and *in vivo* toxicity of respirable tobacco products, and discussed the advantages, disadvantages, and challenges that need to be considered to improve comparing the studies and data reporting for regulatory agencies. Despite these crucial topics that were addressed, there are some limitations to this review, which include that no meta-analysis was performed and a systematic review was not conducted, as this is a narrative review on the focused topic of challenges related to the toxicity assessment of emerging inhalable tobacco products. Thus, it is important to bear in mind that the aims and the scope of this narrative review were to summarize the current research methods and provide insights into how to overcome challenges inherent to these methods from a regulatory standpoint. Addressing these challenges will help generate an initial set of toxicological assessment standards for premarket authorization (PMTA) of emerging tobacco products, which currently lacks scientific standardization.

## Data Availability

Data sharing is not applicable to this article as no new data were created.
